# Perspectives and experiences of youth and caregivers on screening and monitoring islet autoimmunity

**DOI:** 10.3389/fendo.2026.1956622

**Published:** 2026-10-19

**Authors:** Mia Majstorovic, Kelly J. McGorm, John M. Wentworth, Tony Huynh, Fergus Cameron, Maria E. Craig, Elizabeth A. Davis, Kirstine J. Bell, Georgia Soldatos, Megan A.S. Penno, Alyssa Sawyer, Melissa Oxlad, Jennifer J. Couper

**Affiliations:** 1 School of Psychology, Adelaide University, Adelaide, SA, Australia; 2 Adelaide Medical School, Adelaide University, Adelaide, SA, Australia; 3 Endocrinology and Diabetes Department, Women’s and Children’s Hospital, Adelaide, SA, Australia; 4 Department of Diabetes and Endocrinology, Royal Melbourne Hospital, Parkville, Melbourne, VIC, Australia; 5 Department of Endocrinology and Diabetes, Queensland Children’s Hospital, South Brisbane, QLD, Australia; 6 Children’s Health Research Centre, Faculty of Health, Medicine, and Behavioural Sciences, The University of Queensland, Brisbane, QLD, Australia; 7 Department of Endocrinology and Diabetes, Royal Children’s Hospital, Parkville, Melbourne, VIC, Australia; 8 Institute of Endocrinology and Diabetes, Children’s Hospital at Westmead, Westmead, Sydney, NSW, Australia; 9 Department of Endocrinology and Diabetes, Perth Children’s Hospital, Perth, WA, Australia; 10 Charles Perkins Centre and Faculty of Medicine and Health, The University of Sydney, Sydney, NSW, Australia; 11 Diabetes and Vascular Medicine Unit, Monash Health, Melbourne, VIC, Australia

**Keywords:** Australia, early-stage type 1 diabetes, islet autoantibodies, mass screening, monitoring, patient care, qualitative research, type 1 diabetes

## Abstract

**Aims:**

Screening and monitoring programs for islet autoantibodies, which signal risk of clinical type 1 diabetes, are being implemented globally. No studies have qualitatively explored youths’ perspectives on islet autoimmunity screening and monitoring, nor integrated their views with those of caregivers.

**Methods:**

We interviewed 35 Australian youth (aged 9–18 years; median age = 11.5 years; *n* = 18 girls) with one or more islet autoantibodies and 49 caregivers of affected youth. Youth had been monitored for approximately 9 years (median). All families, except one, had a relative with type 1 diabetes. Twenty-one (42.86%) caregivers lived with clinical type 1 diabetes. Data were analysed using reflexive thematic analysis.

**Results:**

Seven themes reflected our findings. Youth had limited understanding of the advantages of monitoring outside of research. They were predominantly focused on testing experiences rather than clinical risk. Caregivers generally displayed strong psychological responses, as did older adolescents. Family experience of diabetes shaped psychological responses in multifaceted ways. Caregivers further emphasised flexible service delivery and specific logistical considerations for screening and monitoring.

**Conclusions:**

Youths’ focus on the practicalities of testing, rather than health risk, is reassuring in the context of planning international screening and monitoring programs. However, it highlights the need to prioritise procedural experiences. Youth should also be educated on the real-world benefits of monitoring. Psychosocial support is needed, particularly for caregivers and older adolescents, with healthcare individualised to the unique influence of family history of clinical diabetes. Caregivers further need reliable, structured monitoring pathways to manage distress, with equity and accessibility prioritised for sustained engagement.

## 1 Introduction

Clinical type 1 diabetes follows a period of months to years after the detection of multiple islet autoantibodies, which signal presymptomatic type 1 diabetes (1–3). Screening for islet autoantibodies enables monitoring of the disease process before insulin initiation (4, 5). Goals are to prevent diabetic ketoacidosis (DKA), facilitate supported education, and enable participation in trials or treatments aimed at preventing or delaying insulin initiation (5, 6). Australian families have participated in screening and monitoring through research initiatives: ENDIA (Environmental Determinants of Islet Autoimmunity; ANZCTRN12613000794707; 7), Type1Screen (ACTRN12620000510943; 8), and the Type 1 Diabetes National Screening Pilot (ACTRN12622000381785; 4).

Islet autoantibody monitoring clinics are being evaluated in Australia and internationally to provide care to children identified through familial and population screening (4, 9–12). A significant unmet research need, as stated in recent international consensus guidance (5) and a large systematic review (13), is to evaluate youths’ and caregivers’ experiences and psychological responses to participating in screening and monitoring programs for islet autoimmunity (IA). This data is critical for enabling consumer-informed, responsive healthcare programs.

Of the scarce existing literature, most studies have employed quantitative methods and surveys. Children aged 10–13 years in the All Babies in Southeast Sweden population screening program were generally receptive to screening for clinical type 1 diabetes risk (14). Children over 3 years of age in ENDIA (15), and youth involved in the TEDDY Study (16) mostly reported satisfaction with their research experiences. ENDIA caregivers also wanted to support research and monitor their children’s type 1 diabetes progression, but expressed concern about blood testing experiences (15). A North American scenario study further found that caregivers prioritised monitoring programs that avoided testing costs and significantly reduced DKA risk, and treatments that delayed insulin dependence (17).

Qualitative research has focused on caregivers in the context of general population screening (18). Findings from the United Kingdom and North America highlight preferences for minimally invasive testing; concerns about the psychological impacts of screening; the importance of healthcare professionals’ support; and issues related to time, convenience, and resources (19–21). Significantly, no qualitative studies have explored youths’ perspectives and experiences of screening and monitoring of IA, despite this gap being recognised as a priority for informing clinical care within the fast-changing international landscape of IA screening and monitoring (5). We present the world’s first integrated qualitative findings from youth (aged 9–18 years) with islet autoantibodies and caregivers of youth with islet autoantibodies to inform the delivery of screening and monitoring services.

## 2 Participants

Youth (aged 9–18 years) identified to have one or more islet autoantibodies through ENDIA, Type1Screen, and one hospital diabetes clinic participated.

The ENDIA pregnancy-birth cohort followed children who had a first-degree relative living with type 1 diabetes for up to 10 years (7). Participants were tested using venous blood draws every 3 to 6 months, according to age. Study visits were conducted face-to-face by research coordinators at metropolitan sites; regional families attended local pathology collection centres. Individuals aged two years or more, with a relative diagnosed with type [MM1.1]1 diabetes, could participate in Type1Screen. Type1Screen monitoring for those identified to have islet autoantibodies was conducted in person and/or via telehealth (8). Those with islet autoantibodies graduating from ENDIA were enrolled in Type1Screen to continue monitoring. Participants with IA had care provided by clinical leads, according to international consensus guidance (2, 5). The same clinicians leading ENDIA also work across Type1Screen.

The minimum age of 9 years was chosen as it coincided around ENDIA study completion, was the average age of Type1Screen participants, and considered developmentally appropriate for interview participation. Youths’ primary caregivers were interviewed, as were the caregivers of children with one or more islet autoantibody aged under 9 years; all were biological parents.

## 3 Materials and methods

### 3.1 Design

To gather rich perspectives, we conducted an in-depth interview study, guided by the Consolidated Criteria for Reporting Qualitative Research (22). We considered a qualitative descriptive design to be best for exploring a topic with limited existing research. This design also enabled us to remain close to participants’ descriptions, using their data, rather than our interpretations, to inform healthcare professionals, policymakers and researchers about families’ perspectives and experiences of IA screening and monitoring.

The Women’s and Children’s Hospital in Adelaide, South Australia provided ethics approval (2024/HRE00146). The study was conducted according to the Declaration of Helsinki and other relevant regulations and institutional guidelines. Participants who provided informed written and verbal assent or consent were interviewed between March 2025 and February 2026.

### 3.2 Recruitment

We purposively sampled participants based on factors in the Results (see Section 4 and **Tables 1**, **2**). These factors included youths’ ages and genders, the screening/monitoring cohort in which families engaged, and their state or territory of residence. KJM and research coordinators, along with clinicians from monitoring clinics, approached families (via email, telephone, and face-to-face). Other recruitment strategies included social media posts and study newsletters.

**Table 1:** Youth characteristics (*n* = 35).

Characteristic	n (%)/median months (IQR)
Gender	
Girl	18 (51.4)
Boy	17 (48.6)
Cohort	
ENDIA	14 (40.0)
Type1Screen	13 (37.1)
ENDIA Graduates in Type1Screen	7 (20.0)
Clinic Referral	1 (2.9)
Monitoring duration	
ENDIA	107 (1-5)
Type1Screen	15.5 (1-7.75)
ENDIA Graduates in Type1Screen	134 (0.5-10)
Clinic Referral	Unknown
State/territory	
South Australia	11 (31.4)
New South Wales	10 (28.6)
Victoria	6 (17.1)
Queensland	5 (14.3)
Western Australia	1 (2.9)
Tasmania	1 (2.9)
Northern Territory	1 (2.9)
Geographical location	
Metropolitan	31 (88.6)
Regional	4 (11.4)
Proband	
Sibling(s)	13 (37.1)
Mother	8 (22.9)
Father	7 (20.0)
Sibling(s) and Mother	2 (5.7)
Sibling(s) and Father	2 (5.7)
Cousin(s)	1 (2.9)
Grandmother, Mother and Sibling(s)	1 (2.9)
Not Applicable	1 (2.9)
Islet autoantibody status	
Multiple	14 (40.0)
Single	12 (34.3)
Transient/Fluctuating Single	7 (20.0)
Progressed to Clinical Type 1 Diabetes	2 (5.7)

**Table 2:** Caregiver characteristics (*n* = 49).

Characteristic	n (%)
Gender	
Woman	43 (87.8)
Man	6 (12.2)
Personal type 1 diabetes diagnosis	
No	28 (57.1)
Yes	21 (42.9)
Child’s islet autoantibody status	
Multiple	19 (38.8)
Transient/Fluctuating Single	13 (26.5)
Single	12 (24.5)
Transient/Fluctuating Multiple	3 (6.1)
Progressed to Clinical Type 1 Diabetes	2 (4.1)
Cohort	
ENDIA	24 (49.0)
Type1Screen	18 (36.7)
ENDIA Graduates in Type1Screen	6 (12.2)
Clinic Referral	1 (2.0)
State/territory	
New South Wales	17 (34.7)
South Australia	12 (24.5)
Queensland	9 (18.4)
Victoria	8 (16.3)
Western Australia	1 (2.0)
Tasmania	1 (2.0)
Northern Territory	1 (2.0)
Geographical location	
Metropolitan	42 (85.7)
Regional	7 (14.3)
Postcode IRSAD^a^ score	
5 (Advantaged)	20 (40.8)
4	10 (20.4)
3	9 (18.4)
2	6 (12.2)
1 (Disadvantaged)	4 (8.2)
Education (highest)	
Tertiary (Bachelor/Honours)	23 (46.9)
Vocational/Trade Certification	10 (20.4)
Tertiary (Masters)	10 (20.4)
Tertiary (Doctorate)	3 (6.1)
Year 12 or Equivalent	2 (4.1)
Not Provided	1 (2.0)
English as a second language^b^	
No	45 (91.8)
Yes	4 (8.2)

^a^ IRSAD, Index of Relative Socioeconomic Advantage and Disadvantage (IRSAD); 1 (most disadvantaged) to 5 (most advantaged). ^b^English as a Second Language status indicates cultural and linguistic diversity without collecting race/ethnicity data.

### 3.3 Data collection

We developed interview guides for youth and caregivers, adapted based on age and emergent interview topics (see Supplementary Material). Seven ENDIA caregivers with lived experience of type 1 diabetes reviewed the guides, which were piloted with an ENDIA family who recommended simplifying questions for younger youths’ comprehension.

MM, a female psychology post-doctoral researcher with qualitative research training, conducted the interviews. All but four interviews were over Zoom. Caregivers mostly interviewed without their child present to share freely. Due to young age, only 5 of 35 youth interviewed without a caregiver present; parents were requested to interject only if the child became emotional or requested assistance. To foster rapport with youth (aged 9–13 years), MM sent a pre-interview introduction about herself and employed youth-friendly online backgrounds.

We considered the sample size appropriate for the research aim(s), method of data collection, and diversity of participants and data (23). Interviews were audio-recorded, transcribed verbatim, and de-identified, then returned to participants for review; we do not know whether youth reviewed transcripts independent of their caregivers. Two caregivers removed potentially self-identifying information. Youth spoke for an average of ≈14 minutes (range: 7–25 minutes); caregivers spoke for ≈23 minutes (range: 13–55 minutes).

### 3.4 Data analysis

We iteratively analysed the data using inductive reflexive thematic analysis (24). Following initial data familiarisation, MM coded the data in NVivo15 and produced preliminary themes. MM refined the themes with MO and KJM. MM emailed the themes to participants for member reflection; one caregiver responded and offered endorsement. All authors approved the final themes.

### 3.5 Reflexivity

We do not have personal experience of type 1 diabetes, screening, or monitoring. As such, we may have approached the research less emotively. MM, who is not a healthcare professional, maintained an audit trail to reflect on how her positionality influenced research processes. The broader authorship team comprises a diverse group of researchers and clinicians from fields including paediatric and adult endocrinology, nursing, clinical and health psychology, and dietetics. KJM, JMW, TH, FC, MEC, EAD, GS, and JJC, but not MM, may have been known to participants from ENDIA, Type1Screen, and/or monitoring clinics.

## 4 Results

We interviewed 84 participants (see **Tables 1**, **2**). Thirty-five youth (*n* = 18 girls), median age 11.5 years (IQR: 9.63-12.58), had been monitored for a median of 109 months (IQR: 24-117). The median length of time between youths’ most recent monitoring test and interview participation was two months (IQR 1 – 6.75). Of the 49 caregivers (*n* = 43 women), 36 cared for youth aged ≥ 9 years, and 13 for youth aged < 9 years. All but one family had relative(s) with type 1 diabetes.

### 4.1 Thematic summary

Seven themes depicted families’ perspectives and experiences of screening and monitoring IA. Below exemplar quotes, we signify caregivers of older (O; ≥ 9 years) and younger (Y; < 9 years) youth. We also specify the cohort in which participants were engaged and youths’ ages.

#### 4.1.1 Theme 1: perceived benefits of participating in screening and monitoring differ

Youth and caregivers may have different motivations and levels of contemplation regarding the advantages of participating in screening and monitoring. Youth mostly emphasised “helping.”

*“… I liked it [participating in monitoring] because I knew that in the future it would be a lot easier to make cures and stuff for diabetes. So other kids, it’d be a lot easier for them…”* (Youth-11, aged 11 years, ENDIA)

While caregivers reiterated this sentiment on behalf of their youth and themselves, some said their interest in screening and monitoring was mainly personal rather than altruistic. They emphasised DKA avoidance and experiencing smoother transitions to diabetes as benefits of monitoring. Regarding youths’ understanding of monitoring, a 12-year-old said monitoring might benefit them, but was uncertain how. Few youth (aged 12–18 years) expressed a positive health outlook toward engagement in monitoring.

*“… if you didn’t monitor, maybe I would have just gone really sick one day, and we wouldn’t know how it happened or when it was growing and what we could have done before…”* (Youth-13, aged 12 years, ENDIA)

Some caregivers and youth (aged 13–18 years) were interested in screening and monitoring because of opportunities to participate in trials aimed at preventing or delaying insulin initiation. Caregivers questioned the value of monitoring without interventions and voiced concerns about trial eligibility/suitability related to age, autoantibody status, and residence. However, they also said that their youths’ willingness to participate and clinical risk would influence their decision-making regarding interventions.

#### 4.1.2 Theme 2: screening and monitoring require clear processes and pathways

Caregivers desired reliable yet tailored screening and monitoring pathways. Caregivers desired electronic booking systems, reminders, and sample receipt and processing notifications. Effective organisation of sample collection from regional areas was also highlighted. Caregivers and an 18-year-old described the importance of timely autoantibody results, with delays causing anxiety and/or annoyance. Caregivers sought a plan for when and how autoantibody results would be delivered to minimise unexpected news and distress.

*“…you just get the call, and you randomly answer it, and one time I was in the carpark at the playground – and you kind of just get dumped with some news…”* (Caregiver-13-Y, ENDIA)

While not discussed by youth, caregivers wanted a clear monitoring pathway following autoantibody detection, including a timeline for consistent testing and appointments.

*“… It was really just we’re going to drop some really [use of profanity] news on you, and then we’ll get back to you at some point. There were conversations between me and [other caregiver], saying, “Where are we at with this? What’s going on? Have they called?.”* (Caregiver-23-O, Type1Screen)

Youth did not share views about their preferred healthcare professionals. Caregivers wanted experts, rather than general practitioners, to deliver IA care. Some sought a multidisciplinary approach that included doctors, diabetes educators, dietitians, and/or mental healthcare professionals. Fewer caregivers were satisfied with medical input alone or minimal healthcare engagement following autoantibody confirmation, viewing it as unnecessary.

*“… the more hands-off approach, the better, because sometimes these sort of engagements are, it’s hard to see what the actual point is. They can just SMS us the results…”* (Caregiver-10-O, ENDIA)

Moreover, caregivers sought communication between IA monitoring and clinical diabetes services to facilitate smoother pathways following clinical diagnosis. Coordination between research and healthcare services nationwide was also desired.

#### 4.1.3 Theme 3: reducing barriers to monitoring through convenience and accessibility

Caregivers explained that work, caring responsibilities, and their youths’ school and extracurricular activities were barriers to participating in both monitoring and trials. Two youth (aged 11 and 12 years) mentioned difficulties scheduling appointments or tests outside of school hours, noting that rural pathology clinics were closed. Caregivers valued accessible parking, flexible sample collection and appointment times, and same-day multidisciplinary services.

*“… [hypothetically] the next day you have to go get blood tests or another day you have to go and see a counsellor or a dietitian or something like that, like when you have to keep taking time out of a day to segregate seeing all those health professionals, that’s when it becomes a bit of a too hard basket…”* (Caregiver-2-Y, ENDIA)

Youth (aged 9–18 years), like caregivers, reported comfort with telehealth, citing convenience and easy access to experts. Caregivers and two youth (aged 9 and 16 years) raised concerns about access to monitoring for rural and regional families. Caregivers recommended telehealth use for such families and those without a nearby clinic, but local clinicians were also desired.

*“…we would need that contact out here [regionally] just for someone aware of it, and then we knew who to go see, when to see them, and what the next step was in terms of monitoring…”* (Caregiver-6-Y, ENDIA)

Other youth (aged 9–18 years) preferred in-person appointments, finding face-to-face interaction easier – an opinion shared by caregivers on behalf of their youth and themselves. Some caregivers desired in-person discussions for matters perceived as serious, such as autoantibody results or clinical diabetes. Additionally, a couple of caregivers and an 18-year-old indicated financial or pragmatic considerations regarding continuous glucose monitors (CGM), with access restricted to research.

#### 4.1.4 Theme 4: psychological responses to the prospect of clinical type 1 diabetes vary

##### 4.1.4.1 Subtheme 1: youth’s neutrality about islet autoimmunity

Younger youth were generally unconcerned about IA, but may worry about clinical diabetes. Only one 9-year-old expressed concern about having autoantibodies. Older youth (aged 11–16 years) also described feeling generally okay or unbothered about their autoantibody results.

*“… I wasn’t really nervous because I’m like, this doesn’t necessarily mean it’s going to give me type 1 diabetes, and if it does, we have heaps of new technology that’ll help … and it’s not actually really too hard to deal with, and it doesn’t actually prevent you from doing sport or anything…”* (Youth-8, aged 12 years, ENDIA)

However, two 18-year-olds reported strong reactions to IA, including fear, uncertainty about disease progression, difficulty coping with the lack of a treatment regimen, and confusion regarding whether they had a medical condition.

*“…until things start happening, I think it’s quite hard just waiting … there’s nothing really out there for the waiting game…”* (Youth-20, aged 18 years, Type1Screen)

When imagining how they would feel if they developed diabetes, youth (aged 9–12 years) described expecting to feel upset initially, but being able to adjust. They imagined feeling upset as they would need to undergo testing, use equipment, navigate management and food changes, face symptoms or medical episodes, and/or experience social life impacts.

*“… I couldn’t have my life as it is now, where I can just go out and just be normal. Not as in you’re not normal, but not worry about what my medical condition is like and what other people are going to think. Hang out with friends – if they’re having food, I’ve got to calculate the carbs…”* (Youth-15, aged 13 years, Type1Screen)

Other youth (aged 9–13 years) anticipated feeling surprised or shocked if they developed diabetes, or could not imagine how they would feel. Caregivers generally thought their youth (aged 9–14 years) were not adversely emotionally affected by IA or the prospect of clinical diabetes, especially when results were presented as low risk. Others considered that their youth (adolescents and a 9-year-old) held varying levels of concern, including about how clinical diabetes could negatively affect their lives.

##### 4.1.4.2 Subtheme 2: caregivers’ intense reactions to youths’ islet autoimmunity

Caregivers described strong emotional responses, including anxiety, worry, shock, devastation, and guilt, in response to their youths’ islet autoantibodies. They described a tension between knowing or not knowing the results.

*“…it’s kind of the damned if you do, damned if you don’t … Like ignorance is bliss, but also – what’s the other one? Knowledge is power. So, you could argue both sides of being positive and negative for knowing…”* (Caregiver-12-O, ENDIA)

Caregivers described varying responses to the uncertainty of disease risk and progression, including changes in autoantibodies over time and across testing methods. Some caregivers reported struggling with this uncertainty – two wanted a clinical diagnosis to occur to facilitate a course of action. Others accepted or rationalised that disease progression was neither imminent nor guaranteed and was beyond their control.*”…we get the result, and we look at what’s going on, then we just move on because it’s just a matter of nothing we can do. We’re just at the mercy of the potential onset of this disease…”* (Caregiver-28-O, Type1Screen)Other caregivers described (hyper)vigilance for clinical diabetes. Factors reportedly linked with heightened distress included higher autoantibody risk, having more than one youth with IA, and youth having additional autoimmune diagnoses.

*“…[the doctor] had said, “Her thyroid’s out, it looks like she might have Graves’ Disease, she’s got gluten reactive antibodies, it looks like she might be coeliac, and on top of that she’s got … [islet autoantibodies].” So, we got this triple whammy…”* (Caregiver-32-O, Type1Screen)

Conversely, some caregivers explained that monitoring did not (markedly) increase their distress; it provided “control, “ and for some, reduced anxiety or dissipated initial distress about their youths’ IA.

#### 4.1.5 Theme 5: complex influence of family connection to clinical type 1 diabetes

##### 4.1.5.1 Subtheme 1: family connection to type 1 diabetes shapes youths’ perspectives

A family connection to type 1 diabetes may be both helpful and unhelpful for youth psychologically. Both caregivers and youth described youth observing and talking with family member(s) about diabetes, which normalised it. However, youth (aged 9–18 years) who had witnessed their siblings’ type 1 diabetes diagnoses or medical emergencies reported feeling nervous for them or worried about personally developing the condition.

*“… I would feel a little bit nervous because of all the things that my sister’s gone through in her life … she’s been in a Royal Flying Doctor’s aeroplane, and that was scaring me when we were camping…”* (Youth-18, aged 9 years, ENDIA)

Two caregivers understood their youths’ concerns about developing diabetes due to observing its impact on family members. Other caregivers viewed that an adaptive, accepting family attitude toward, and stable management of diabetes, would positively influence their youths’ perceptions. Both youth and caregivers generally described youth as reassured by family members managing diabetes and knowing they would have their support if they developed the condition.

*“…because my sister has it, then we can both have it … I wouldn’t feel like it’s amazing, I’d just feel like it’s a bit better because I know that she can help me…”* (Youth-19, aged 12 years, ENDIA)

Mostly caregivers suggested that, for their youth, monitoring and the prospect of clinical diabetes engendered a sense of shared experience and family belonging.

##### 4.1.5.2 Subtheme 2: caregivers’ experiences with the condition impact coping

Caregivers with personal or family experience (including with another child) of type 1 diabetes expressed adjusting to their youths’ IA and confidence in coping with their impending diagnosis. Caregivers said awareness of their youths’ predisposition to clinical type 1 diabetes reduced their emotional responses.

*“…it [IA] doesn’t affect me much because you already know the kids’ father got this disease, so you know there’ll be a chance your kids might have it…”* (Caregiver-14-O, ENDIA)

Others with a family history, particularly another child with type 1 diabetes, expressed distress when anticipating or learning about their youth’s IA. Some discussed a sense of urgency.

*“… when our eldest daughter was diagnosed, it’s very, “Quick. Let’s go. You’ve got to get on top of it.” It’s very fast-paced. And to get that phone call [about detectable autoantibodies] throws you back into that mind frame, but then nothing’s happening… “* (Caregiver-24-O, Type1Screen)

Caregivers recalled concerns about their youth developing IA or diabetes leading up to the age at which family members had been diagnosed. Caregivers without diabetes, or speaking on behalf of a partner without the condition, perceived they had a different (outsider or more anxiety-provoking) experience.

#### 4.1.6 Theme 6: making youths’ testing experiences tolerable is critical

##### 4.1.6.1 Subtheme 1: procedures are central to youths’ experiences

Youth (aged 9–18 years) expressed discomfort and distress related to procedures (e.g., blood and oral glucose tolerance tests). Most preferred finger-pricks over venous blood draws because they were quicker and less painful, as corroborated by caregivers. The anticipation of more tests if youth developed diabetes was said to heighten caregivers’ responses to their youths’ testing experiences.

*“… it added an element of stress and worry about the testing and the blood tests, which I know were all voluntary for us to submit to for [youth’s name], but it added an element of, “Am I harming my child?.”* (Caregiver-9-Y, ENDIA)

Caregivers discussed factors influencing their youths’ testing experiences, including initial blood draw experiences and familiarity with tests, age, temperament, life circumstances, and caregivers’ responses. Youth (aged 9–12 years) similarly mentioned age and the significance of their initial tests.

*“… Caregiver: You were really worried, weren’t you? Youth: Yes, because that was my first blood test, I think it was…”* (Youth-7, aged 9 years, and Caregiver-7-O, Type1Screen)

Other youth described coping well with the tests/equipment and/or adjusting over time. Caregivers additionally discussed how their youth experienced distress, but also adjusted and coped with CGM over the short wear period (10–14 days). Some caregivers thought their youth would be embarrassed if peers noticed their CGM, but youth (aged 10–14 years) communicated being unbothered by this possibility.

*“…Youth: It’d just be like a dot on my arm, like it would not impact my day-to-day life … My shirt would be covering it anyway … Interviewer: How would you feel if people did notice it? Youth: I probably wouldn’t care…”* (Youth-32, aged 14 years, Type1Screen)

When asked about their preferred mode of administration for use in trials, youth (aged 9–14 years) generally said tablets or liquids, emphasising taste and ease of ingestion. When considering trials, caregivers discussed evaluating their youths’ well-being, the suitability of the administration mode for their youths’ abilities, and its invasiveness/intensity. One caregiver expressed that their youth’s aversion to procedures increased their interest in delaying the condition.

##### 4.1.6.2 Subtheme 2: means of supporting youth with procedures

Youth (aged 9–13 years) described methods that helped them with venous blood tests, including distraction techniques, virtual reality headsets, EMLA products, and Buzzy Bee devices, and butterfly needles. One caregiver considered their youth’s blood-taking fear as extreme and contemplated psychotherapy or hypnosis. Other caregivers discussed reducing the frequency of blood tests and valued routine testing processes. Youth (aged 9–18 years) and caregivers further recommended that experienced, consistent individuals perform the tests. They mentioned the benefits of nurses/phlebotomists with paediatric training and gaining youths’ assent for tests. Other caregivers were more insistent.

*“… it’s been quite a rollercoaster ride. We actually used to have to pin her down and physically hold her down to get blood out of her…”* (Caregiver-1-O, ENDIA)

Some youth (aged 9–16 years) praised efforts or had no suggestions to improve testing.

*“… I’ve always disliked the idea of drawing blood and blood tests, but it’s never really been what’s happened, like what the doctors and stuff are doing or what my family has been doing [that has made it more difficult]…”* (Youth-27, aged 12 years, Type1Screen)

Furthermore, youth (aged 9–13 years) described enjoying city appointments and engaging in activities after blood-taking (e.g., getting food, a zoo trip). Others reported going home and/or resting. Youth (aged 9–12 years) looked forward to receiving rewards after testing.

#### 4.1.7 Theme 7: families value supportive relationships

Caregivers emphasised rapport with healthcare professionals, conveying that continuity of care, such as having a primary, reliable nurse or diabetes educator, was valuable. They perceived that rapport with clinicians would ease, or had eased, the transition to clinical diagnosis. Youth liked their interactions with healthcare professionals.

*“… they’re funny, and they’re nice [the nurses]…”* (Youth-30, aged 10 years, ENDIA)

However, two 9-year-olds mentioned discomfort when first meeting healthcare professionals. An 18-year-old said frequent in-person appointments with doctors were “a bit scary, “ but did not specify why [Youth-33]. Youth-24 proposed how to enhance comfort.

*“… knowing their name and knowing what they look like, and their personalities before I meet them…”* (aged 9 years, ENDIA)

Caregivers valued empathy and accessibility from healthcare professionals. They appreciated healthcare professionals delivering autoantibody results sensitively.

*“… it was a long phone call, I had plenty of time to try and process it and ask questions … the staff on the other end were helpful, supportive…”* (Caregiver-18-O, Type1Screen)

Some caregivers desired access to mental healthcare to cope with their youths’ IA. Fewer caregivers suggested their concerns were not amenable to such support or perceived it as unnecessary due to a family history of type 1 diabetes. Some considered such support more necessary after a clinical diagnosis. Minimal caregivers thought mental healthcare may be helpful for their youth, specifically if they were close to needing insulin or had been diagnosed. However, some youth were unsure or did not want additional support.

*“…Interviewer: Is there any help or support you’d like to be receiving from healthcare professionals or anyone else? Youth: No, not really. Feel pretty good…”* (Youth-35, aged 16 years, Type1Screen)

A couple of 18-year-olds requested peer support, while a 14-year-old expressed such interaction would feel “forced” [Youth-32]. Caregivers also suggested peer support for themselves and/or their youth, commenting, though, that younger youth might not understand the purpose.

## 5 Discussion

We present world-first qualitative findings from youth with islet autoantibodies and caregivers of affected youth. Youth and caregivers differed in their priorities: youth were largely altruistic, present-focused, and concerned with the procedures of monitoring, while caregivers emphasised structure, service accessibility, and future-oriented benefits of screening and monitoring. Psychological responses also diverged: youth generally expressed minimal concern about IA and caregivers described intense emotional reactions, though variation was still evident across participants. Below, the themes are considered in terms of their key messages and the implications in practice of those messages.

First, youth, particularly younger youth, expressed willingness to participate in screening and monitoring to help others [also see (14–16)], but showed a generally limited understanding of its benefits to them outside of the research context. Age-appropriate education for youth on the advantages of monitoring in a real-world healthcare context is needed as screening and monitoring programs enter healthcare. Without a clear motivation for their involvement, youth may experience added testing-related distress and sustaining their engagement could be challenging. Reassuringly, caregivers were clearer about the benefits, including DKA avoidance and participation in interventions, aligning with previous reports (5, 17, 19). Engagement in trials and treatments should be communicated upfront as a benefit of screening and monitoring participation and offered to eligible participants as they progress through the monitoring pathway.

Second, youth generally expressed minimal concern about their IA but prioritised their testing experiences. Our finding reinforces the need to apply youths’ and caregivers’ recommendations for improving procedural experiences to minimise youths’ immediate distress [also see (15, 19–21, 25)]. Youths’ sense of neutrality about their IA appears reassuring, though, in the context of the planning of screening and monitoring programs worldwide (5). Youths’ lack of concern could reflect their limited understanding of clinical risk and difficulties forecasting its implications (26–28). As youth age and their understanding develops, modified care will be needed, with our findings suggesting that older adolescents experience distress in response to IA. Further studies should longitudinally quantify youths’ distress with a larger sample to validate this finding.

Further to this point, while we expected caregivers to report adverse psychological reactions to their youths’ islet autoantibodies (29), it is noteworthy that some found monitoring reassuring and anxiety-reducing. Our findings support the need for individualised care that avoids assumptions about caregivers’ psychological states, while ensuring that psychological services are available for those in need. Additionally, our findings indicate that first-degree relatives exhibit *diverse* emotional responses. This finding broadens others’ quantitative studies wherein caregivers with a family history of type 1 diabetes experienced greater anxiety in response to their children’s clinical risk than those without a family history (30, 31). Similar results were identified for children (26). Hence, our findings extend prior work by suggesting that clinicians should consider individuals’ unique experiences and appraisals of diabetes, not only the presence of a family history.

Third, caregivers expressed a desire for structured, predictable, and reassuring screening and monitoring pathways, which is especially important given the uncertainty accompanying the likely time course of metabolic progression (3, 33). One logistical implication is ensuring tight coordination between monitoring clinics and pathology services to streamline processes. While caregivers emphasised structure, healthcare customisation will also be critical, as families expressed varying preferences regarding, for example, the involvement of multidisciplinary professionals and telehealth use. For reliable, equitable service access, we further recommend upskilling diabetes educators, including rurally based educators, to ensure support is available between clinic visits. Emerging IA care models from Western Australia and the United Kingdom will inform implementation alongside our findings (32, 33). Similar aspects include hybrid (telehealth and in-person) consultation and the involvement of experienced clinicians (32, 33). Complementary to efficient, clear service provision, flexible service delivery options will be essential for accommodating families and sustaining their ongoing participation in monitoring.

## 6 Strengths and limitations

This study addresses priority research questions around the impacts of screening and monitoring on young people and the family unit (13). Study strengths were participants being located across most Australian states/territories, including regional settings, and the length of time families were monitored. Given ENDIA’s longitudinal design, families would have received more contact and educational input when compared to Type1Screen participants. The vast amount of data collected during interviews with 84 participants, lasting on average 14–23 minutes, was another strength. However, few older adolescents and fathers were interviewed, almost all families had a first-degree relative with diabetes, and most caregivers had a tertiary education. Wider inclusion of people from diverse sociocultural backgrounds would enhance our ability to understand the responses of a broader sample. Future research involving the experiences of more fathers, and those without lived experience of type 1 diabetes, would also inform programs intended for the general population. Additionally, gender differences in youths’ experiences were beyond the scope of this study and could be considered in future research to enable tailored clinical care.

## 7 Conclusion

Our findings underscore the need to support youth with their immediate experiences of monitoring, whilst responding to caregivers’ future-focused concerns. Youth were predominantly concerned about their testing experiences, rather than their clinical risk and were generally uncertain of the benefits of monitoring. Caregivers focused on youths’ health risk status, associated implications, and the healthcare pathways forward. Participants’ recommendations to enhance youths’ procedural experiences, support families’ psychological health in tailored ways, and improve service organisation and accessibility will guide future screening and monitoring initiatives.

## Data Availability

The raw data supporting the conclusions of this article will be made available by the authors, without undue reservation.

## Group members of ENDIA Study Group

Simon C Barry, Peter Colman, Emma Hamilton-Willliams, Leonard Harrison, Aveni Haynes, Ki Wook Kim, Grant Morahan, William D Rawlinson, Timothy Spelman, Rebecca L Thomson, Jason Tye-Din, and Peter Vuillerman.
