# Supplementary material for: Perspectives and experiences of youth and caregivers on screening and monitoring islet autoimmunity
Source: Front Endocrinol (Lausanne). 2026 Oct 19;17:1956622. doi: 10.3389/fendo.2026.1956622 (PMC13724607; doi:10.3389/fendo.2026.1956622)
Supplement: Supplementary file 1 [file SupplementaryFile1.docx]

**ELECTRONIC SUPPLEMENTARY MATERIAL: INTERVIEW GUIDES**

**Youth Interview Topic Guide**

**Hello! My name is Mia.**

What’s your name? How old are you?

**You took some tests to help us learn about type 1 diabetes. Thank you for being so helpful! The reason for our chat today is to talk a bit about those tests.** Is that okay?

Thank you. I’m going to start recording so I make sure I get what you say right, and then I’ll ask the first question.

1. **What, if anything, do you know about type 1 diabetes?**

PROMPTS:

- Do you talk to anyone about type 1 diabetes? If so, with whom?
- What do you talk about?

2. **Have you heard of** **diabetes antibodies**? If yes, what do you know about diabetes antibodies?
3. **How have you been helping us to learn more about type 1 diabetes?**

PROMPTS

4. **Where do you go to do the tests?**

- How would you feel about talking to someone over the computer (like we’re doing today) instead of going to [X] for the tests? [If applicable]

5. **Whom do you see** **when you go to [X]?**
6. **What happens** **when you go to [X]?**
   a. **How do they collect your blood?**

PROMPTS:

- Needle? Finger prick?
  b. **What, if anything, makes it easier for you?** (What, if anything, do the nurses or doctors do to make things easier for you?)
  c. What, if anything, makes it hurt less/less sore?
  d. **What, if anything, makes it harder for you?** (What, if anything, do the nurses or doctors do that makes things harder for you?)
  e. What, if anything, makes it hurt more/more sore?
  f. **Why do you think they test your blood?**

7. **What else do you do when you go to [X]?**
8. **How do you feel about going to [X] and having the tests?**

PROMPTS:

- Ok? Happy? Sad? Scared? Mad?
- Do you worry about going to see [X]?

9. **What, if anything, do you or your [appropriate caregiver term] usually do after you go to [X] that helps with how you feel?**

PROMPT:

- Do you go somewhere?
- Do you do something you like to do?

10. **What, if anything, could people do to make it better for you when you have these kinds of tests?**
11. **What, if anything, do you like about helping to learn about type 1 diabetes?**
12. **What, if anything, don’t you like about helping to learn about type 1 diabetes?**

The next question might be a bit tricky for some people to answer. Please take your time, and remember, you don’t have to answer if you don’t want to.

13. **Imagine if someone told you that you had type 1 diabetes. How do you think you would feel?**
    a. **What would you think if someone said you had type 1 diabetes?**
14. **Is there anything else you want to tell me about the tests or type 1 diabetes?**

Thank you so much for talking to me! Our chat is going to help people like me improve things for young people who take tests, as you did.

Thanks again. I’m going to talk to your [applicable caregiver term] now.

**Caregiver Interview Topic Guide**

### **Introduction**

Thank you so much for letting me interview your child [if applicable].

### **Reflections from the child’s interview [if applicable]**

### You’ve listened to your child talk about their experiences with screening and/or monitoring for early-stage type 1 diabetes.

- What thoughts or reflections, if any, would you like to share about what they’ve said?

PROMPT:

- Did any of what they shared surprise you?

**Thoughts on the screening and monitoring process**

- What, if anything, has worked well during your child’s participation in screening and/or monitoring for early-stage type 1 diabetes?
- What, if anything, has not worked well during your child’s participation in screening and/or monitoring for early-stage type 1 diabetes?
- What would a “family-friendly” approach to screening and monitoring look like for you and your family?

PROMPTS:

- What do you think about screening and/or monitoring taking place over telehealth? How, if at all, might that setup affect your family?
  - How would you feel about a hybrid (telehealth and in-person) approach?

### **Impacts of participating**

- How do you think your child feels about taking part in screening and/or monitoring for type 1 diabetes?

PROMPT:

- How do you think your child feels about the tests and procedures involved (PROMPT, if necessary: e.g., blood tests, finger pricks, throat/nose swabs, OGTT, CGM, etc.)?
- What changes, if any, have you noticed in your child because of their participation in screening and/or monitoring for type 1 diabetes?
- What, if anything, do you think could be done to make the screening and/or monitoring process easier/better for your child?
- What changes, if any, have occurred for you because of your family’s participation in screening and/or monitoring for type 1 diabetes?
- What changes, if any, have you noticed in other family members because of your family’s participation in screening and/or monitoring for type 1 diabetes?

PROMPT:

- What, if anything, could be done to make the screening and/or monitoring process easier/better for you and other family members?

### **Anxiety and worry**

- How, if at all, has your family’s participation in screening and/or monitoring for type 1 diabetes affected your level of worry about your child’s health?

PROMPTS:

- What, if any, support approaches have proven useful?
- What, if any, support approaches have proven less useful?
- How, if at all, has your family’s participation in screening and/or monitoring for type 1 diabetes affected your level of concern about your child’s risk of developing type 1 diabetes?

PROMPT:

- What, if anything, has helped to manage any worry you have about your child’s risk of developing type 1 diabetes?
- How, if at all, has your behaviour towards your child changed after receiving the antibody test results?
- What lifestyle changes, if any, have you made for your child after receiving the antibody test results?

**Understanding and education**

- What do you know about type 1 diabetes?
- What do you know about diabetes antibodies (we call them islet autoantibodies)?
- How have your child’s islet autoantibody results been explained to you?
- PROMPTS:
- What do you think about the information and/or support you received?
- Which healthcare professionals, if any, would you like to be involved in the follow-up process? What kind of support, if any, would you like to receive from them?
- What other information, if any, would you have wanted to receive before engaging in screening and monitoring for type 1 diabetes?

**Ongoing islet autoantibody monitoring**

- Have you considered continuing to have your child monitored for islet autoantibodies?
- Why/why not?

**Future research participation**

If your child were eligible, would you be interested in participating in clinical trials to prevent or delay type 1 diabetes?

- Why yes/why not?

**Demographic Information**

- What is your highest education level?
- Is English your first language?

### Thank you!

That’s all the questions I have for you. Is there anything else you’d like to share with me about screening and/or monitoring for early-stage type 1 diabetes or type 1 diabetes?

**Wrap Up**

Thank you so much for talking to me. From here, I will make a transcript of the interview and send you a copy so that you can make sure it’s an accurate reflection of our discussion.

When we have some themes and findings to share, we will also send these to you for your feedback. Thanks again for your time.

***NOTE: Participants were asked numerous questions to inform a larger study. Not all questions and responses are relevant to this manuscript.***
